# Surface-Attached Poly(oxanorbornene) Hydrogels with Antimicrobial and Protein-Repellent Moieties: The Quest for Simultaneous Dual Activity

**DOI:** 10.3390/ma11081411

**Published:** 2018-08-11

**Authors:** Monika Kurowska, Vania Tanda Widyaya, Ali Al-Ahmad, Karen Lienkamp

**Affiliations:** Freiburg Center for Interactive Materials and Bioinspired Technologies (FIT) and Department of Microsystems Engineering (IMTEK), Albert-Ludwigs-Universität, Georges-Köhler-Allee 105, 79110 Freiburg, Germany; monika.kurowska@imtek.uni-freiburg.de (M.K.); vania.widyaya@imtek.uni-freiburg.de (V.T.W.); ali.al-ahmad@uniklinik-freiburg.de (A.A.-A.)

**Keywords:** antimicrobial polymer, coatings, hydrogel, protein-repellent polymer, surface-attached polymer network

## Abstract

By copolymerizing an amphiphilic oxanorbornene monomer bearing N- tert-butyloxycarbonyl (Boc) protected cationic groups with an oxanorbornene-functionalized poly(ethylene glycol) (PEG) macromonomer, bifunctional comb copolymers were obtained. Varying the comonomer ratios led to copolymers with PEG contents between 5–25 mol %. These polymers were simultaneously surface-immobilized on benzophenone-bearing substrates and cross-linked with pentaerythritoltetrakis(3-mercapto­propionate). They were then immersed into HCl to remove the Boc groups. The thus obtained surface-attached polymer hydrogels (called SMAMP*-*co*-PEG) were simultaneously antimicrobial and protein-repellent. Physical characterization data showed that the substrates used were homogeneously covered with the SMAMP*-*co*-PEG polymer, and that the PEG moieties tended to segregate to the polymer–air interface. Thus, with increasing PEG content, the interface became increasingly hydrophilic and protein-repellent, as demonstrated by a protein adhesion assay. With 25 mol % PEG, near-quantitative protein-adhesion was observed. The antimicrobial activity of the SMAMP*-*co*-PEG polymers originates from the electrostatic interaction of the cationic groups with the negatively charged cell envelope of the bacteria. However, the SMAMP*-*co*-PEG surfaces were only fully active against *E. coli*, while their activity against *S. aureus* was already compromised by as little as 5 mol % (18.8 mass %) PEG. The long PEG chains seem to prevent the close interaction of bacteria with the surface, and also might reduce the surface charge density.

## 1. Introduction

Bacterial infections related to the use of medical devices is one of the main causes of nosocomial infections [1], and might cost the lives of up to 10 million people worldwide every year by 2050 if current trends continue [2]. Thus, numerous strategies to prevent biofilm formation have been reported in the past two decades [3,4,5,6,7,8,9,10,11,12,13,14,15,16,17,18,19]. One prominent strategy is to coat materials with polycationic surfaces which then kill bacteria upon contact due to their interaction with the bacteria's negatively charged cell membranes [9,20,21]. The exact mechanism of this interaction is still unclear, however it seems as if charge density plays an important role to either damage the bacterial membrane or to pin the bacteria to the surface and thereby prevent their proliferation [21,22,23]. However, it was also shown that cationic polymer surfaces get contaminated by the debris of dead bacteria, and therefore they do not prevent bacterial biofilm formation on the long run [24,25]. A number of studies have addressed this problem by combining antimicrobial and protein-repellent moieties in one material. This has been nicely reviewed by Chen [26]. For example, Paris et al. grafted the antimicrobial peptide nisin onto surface-immobilized anti-adhesive anionic hyaluronic acid [27]. Yang et al. coupled antibacterial chitosan to protein-repellent poly(2-hydroxyethyl methacrylate) polymer brushes [28]. Ye et al. presented a membrane that was surface-functionalized with block copolymer brushes containing non-fouling polyzwitterionic moieties and antimicrobial quaternary ammonium parts [29]. In these and other examples, some degree of simultaneous dual antimicrobial activity and protein-repellency was obtained, yet they also demonstrate that the integration of anti-adhesive and antibacterial components into one surface coating without loss of one of the two functionalities remains a challenge [30]. We also previously reported the integration of polymeric synthetic mimics of antimicrobial peptides (SMAMPs) and protein-repellent polyzwitterions, either side-by-side using micro-/nanostructuring approaches [31,32], or hierarchically using a grafting-onto approach [31]. However, these were either obtained via complicated, multi-step procedures (which is undesirable for technical applications) [31,32], and/or the surface modification could not be sufficiently controlled, so that no clear structure–property relationships were obtained [31]. 

**Scheme 1:**
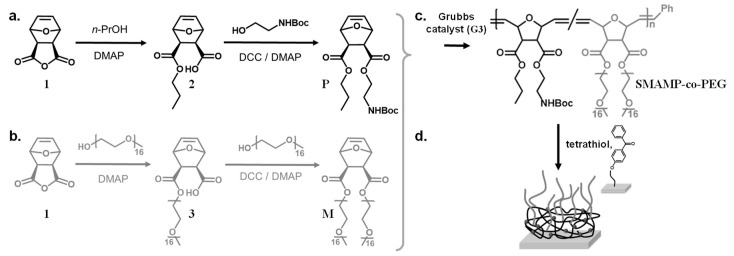
Synthesis of the protected propyl monomer P (**a**) and the PEG macromonomer M (**b**). Copolymerization of P and M with different mass ratios gave the copolymer SMAMP-*co*-PEG (**c**). Using UV irradiation, the copolymers were simultaneously surface-immobilized on a substrate pre-treated with a benzophenone linker, and cross-linked using the tetrathiol cross-linker (**d**).

We here present a bifunctional comb copolymer consisting of antimicrobial, polycationic SMAMP moieties and protein-repellent poly(ethylene glycol) (PEG) side chains (Scheme 1). SMAMPs are well known for their high antimicrobial activity combined with good cell compatibility [33], yet they are also protein-adhesive due to their cationic nature [25]. PEG, on the other hand, is well known for its protein-repellency [17,34]. The aim of this study was to investigate the structure–property relationships of such SMAMP-PEG copolymers, and to see whether simultaneously bifunctional materials could be obtained using this synthetic platform. Importantly, the PEG moieties were obtained through a macromonomer, so that the resulting material had a hierarchical structure, with PEG extending over the SMAMP moieties. The question was how much PEG was needed to sufficiently shield the SMAMP moieties from proteins, yet still retain the antimicrobial activity. For this, copolymers with varying SMAMP and PEG content (5, 10, and 25 mol % PEG, named SMAMP*-*co*-X%PEG, where X refers to the PEG content) were synthesized and surface-immobilized, and their physical properties and bioactivity were evaluated and compared. 

## 2. Experiment

### 2.1. Materials

All chemicals were obtained as reagent grade from Sigma-Aldrich (Taufkirchen, Germany), Carl Roth (Karlsruhe, Germany), Fluka (Taufkirchen, Germany), or Alfa Aeser (Karlsruhe, Germany), and used as received. High performance liquid chromatography (HPLC) grade solvents were purchased dry from Carl Roth (Karlsruhe, Germany), and used as received. Dichloromethane (DCM) was freshly distilled over P_2_O_5_ before use.

### 2.2. Instrumentation.

Gel permeation chromatography (GPC, in THF, calibrated with poly(styrene) standards) was performed on a PSS SDV column (PSS, Mainz, Germany). NMR spectra were recorded on a Bruker 250 MHz spectrometer (Bruker, Madison, WI, USA). MALDI-TOF mass spectra were measured on Bruker Autoflex III TOF/TOF mass spectrometer (Bruker, Billerica, MA, USA) equipped with a 200 Hz beam laser. The measurement was performed by using trans-2-[3-(4-tert-butylphenyl)- 2-methyl-2-propenylidene]malononitrile (DCTB) as matrix and Na^+^ as ionization agent in CHCl_3_. Measurements were done at the Institute for Macromolecular Chemistry of the University of Freiburg. The thickness of the dry polymer layers on silicon wafers was measured with the auto-nulling imaging ellipsometer Nanofilm EP3 (Nanofilm Technologie GmbH, Göttingen, Germany), which was equipped with a 532 nm solid-state laser. For each sample, the average value from three different positions was taken. The irradiation of samples with UV light was conducted using a BIO-LINK Box (Vilber Lourmat GmbH, Eberhardzell, Germany) with different wavelengths (254 and 365 nm). Attenuated total reflection Fourier transform infrared spectroscopy (ATR-FTIR) spectra were recorded from 4000 to 400 cm^−1^ with a Bio-Rad Excalibur spectrometer (Bio-Rad, München, Germany), using a spectrum of the blank double side polished silicon wafer as background. Double side polished silicon wafers were used as substrates for the FTIR experiments. Contact angles were measured on an OCA 20 system (Dataphysics GmbH, Filderstadt, Germany). The average value of the contact angles was calculated from four measurements per sample. The topography of the surfaces was imaged with a Dimension Icon atomic force microscope (AFM) (Bruker). Commercial Bruker ScanAsyst Air cantilevers (length: 115 µm; width: 25 µm; spring constant: 0.4 Nm^−1^; resonance frequency: 70 kHz) were used. All AFM images were recorded in the ScanAsyst mode in air, respectively. The obtained images were analyzed and processed with the software ‘Nanoscope Analysis 9.1’. For each sample, the root mean square (RMS) average roughness from three images of an area of 5 × 5 µm² at different positions was taken. Photoelectron spectroscopy (XPS) data was obtained from on a Perkin Elmer PHI 5600 ESCA System (PerkinElmer, Waltham, MA, USA). The X-ray source was a Mg anode with an energy of 1253.6 eV. The aperture size was 400 µm, the angle was 45°. The typical measurement size is 10 µm^2^. Samples were measured at room temperature. Surface plasmon resonance spectroscopy (SPR) experiments were performed on a RT2005 RES-TEC device in Kretschmann configuration from Res-Tec, Framersheim, Germany. Excitation was done with a He-Ne-Laser with λ = 632.8 nm. SPR substrates were homemade (LaSFN9 glass from Hellma GmbH, Müllheim, Germany; coated with 1 nm Cr and 50 nm Au at the Cleanroom Service Center (RSC) of the Department of Microsystems Engineering, University of Freiburg, using the device CS 730 S (Von Ardenne, Dresden, Germany). The set-up and measuring procedures of the kinetics experiments and the full angular reflectivity scans have been reported previously [25]. Experiments to test the antimicrobial activity of the polymer networks were performed using a previously described spray assay, which is a modification of the Japanese Industrial Standard JIS Z 2801:2000 “Antibacterial Products Test for Anti-bacterial Activity and Efficacy”, and was reported previously [35]. *S. aureus* (ATCC29523) and *E. coli* (ATCC25922) were used. 

### 2.3. Synthesis

Monomers—The synthesis and characterization of the monomers P and M was described previously [36,37]. 

Copolymerization—Polymerizations were performed under nitrogen using standard Schlenk techniques. The respective amounts of propyl SMAMP and PEG monomers were dissolved in anhydrous THF (5 mL for SMAMP-*co*-5%PEG and SMAMP-*co*-10%PEG copolymers, and 6 mL for SMAMP-*co*-25%PEG copolymer). The Grubbs third generation catalyst was dissolved separately in 2 mL anhydrous THF and added to the monomers solution in one shot. After 40 min stirring, the polymerization was quenched by adding 1 mL (750 mg, 10 mmol) ethyl vinyl ether. The mixture was stirred for 30 min. The solvent was removed under reduced pressure and the crude polymer was purified by precipitation into a cold diethyl ether/n-hexane mixture, yielding white solid. Yield: 80%. The reagent amounts for each copolymer are included in Table 1. The SMAMP reference polymer was synthesized as reported previously [33].

Surface anchor groups and surface functionalization—The molecule used to covalently bind the SMAMP-*co*-PEG copolymers to the Si surfaces was a benzophenone-functionalized triethoxysilane (3EBP); a lipoic acid disulfide (LS-BP) was used for the gold substrates for the surface plasmon resonance spectroscopy (SPR) experiments. LS-BP and 3EBP-silane were synthesized as described previously; the surfaces were also functionalized as described previously [25,33]. 

Surface-attached polymer networks. A stock solution (Solution A) was prepared by dissolving pentaerythritol-tetrakis-(3-mercaptopropionate) (1 mL, 1.3 g, 2.6 mmol) in THF (50 mL). SMAMP-*co*-5%PEG copolymer (10 mg, 0.02 mmol), SMAMP-*co*-10%PEG (11 mg, 0.02 mmol), or SMAMP-*co*-25%PEG (14 mg, 0.02 mmol) were dissolved in Solution A (0.25 mL). Chloroform (0.8 mL) was added as co-solvent. The mixture was stirred for 60 s. From this solution, a polymer film was spin cast onto a 3-EBP treated silicon wafer or a LS-BP treated gold substrate at 3000 rpm for 30 s. The resulting polymer film was cross-linked at 254 nm for 30 min. It was then washed with THF to remove unbound polymer chains and dried under N_2_-flow. To remove the Boc protective groups, the film was immersed in HCl (4 M in dioxane) for 12 h and washed twice with ethanol, and dried under N_2_-flow. 

Physical and biological characterization—All experiments were performed as reported previously for other surface-attached polymer networks [25].

## 3. Results and Discussion

### 3.1. Material Design

The two above described functionalities were combined by copolymerizing the oxanorbornene-functionalized PEG macromonomer M with the oxanorbornene monomer P via ring-opening metathesis polymerization (ROMP, Scheme 1). The P moieties carry each a propyl group and a N-Boc protected primary amine group, which would be cationic after removal of the Boc group. This would impart antimicrobial activity onto the polymer upon deprotection. The thus obtained N-Boc-protected SMAMP-*co*-PEG copolymers were simultaneously surface-attached and cross-linked by UV-activated thiol-ene reactions with pentaerythritoltetrakis(3-mercapto­propionate) and the polymer double bonds, and by C,H insertion reactions between the benzophenone groups on the substrate and polymer CH-bonds (Scheme 1). After surface-attachment and deprotection (giving SMAMP*-*co*-PEG surfaces), the cationic moieties of the SMAMP* repeat units and the PEG grafts microphase separated. Thus, by varying the PEG content of the copolymer, the domain sizes and the content of PEG at the polymer–air interface could be controlled, so that materials with simultaneous protein repellency and antimicrobial activity were obtained, as described in detail below.

### 3.2. Monomer Synthesis

The protected cationic monomer P and the PEG macromonomer M were each synthesized in a two-step reaction following the procedures described in the literature (Scheme 1a,b) [36,38]. To synthesize P, the oxanorbornene anhydride 1 was ring-opened by 1-propanol in the presence of catalytic amounts of a base (*N,N*-dimethylaminopyridine, DMAP), so that the propyl ester 2 was obtained. In the second step, the remaining carboxyl functionality was esterified with 2-(N-Boc)aminoethanol using standard peptide coupling conditions (DMAP and dicyclohexylcarbodiimide, DCC, Scheme 1a) [33,37]. In a similar reaction sequence, anhydride 1 was ring-opened by an ω-methoxy PEG alcohol with 16 repeat units (average molecular weight *M_n_* = 750 g mol^−1^_,_ Scheme 1b) using DMAP as base; the second PEG chain was attached to the carboxyl group of intermediate 3 using DMAP/DCC, so that the symmetrical macromonomer M was obtained (Scheme 1b). The ^1^H-NMR spectra of the monomer P and the macromonomer M obtained after monomer purification matched the literature data [36,38]. In particular, the NMR spectrum of macromonomer M, with less peaks than the spectrum of intermediate 3, indicated that a symmetrical two-armed compound was obtained (Figure S1a). Both the macromonomer M and the intermediate 3 were analyzed by gel-permeation chromatography (GPC, in THF, Figure S1b). In the GPC elugram, both M and 3 had a single peak with a low polydispersity index (*M_w_*/*M_n_* = 1.04). The macromonomer M eluted at shorter retention times than the intermediate 3, i.e., it had a higher molar mass. Notably, there was no peak at the elution time of the intermediate, indicating that a quite pure macromonomer was obtained. Using a calibration curve (polystyrene standards), the number average molar mass *M_n_* of M was calculated as 1920 g mol^−1^, and that of 3 as 960 g mol^−1^. Both numbers are in good correlation with the expected masses (=oxanorbornene head group + PEG residues). The structure of the macromonomer was further confirmed by MALDI-TOF mass spectrometry (Figure S1c). All these findings matched literature reports [36].

### 3.3. Polymer Synthesis 

**Figure 1:**
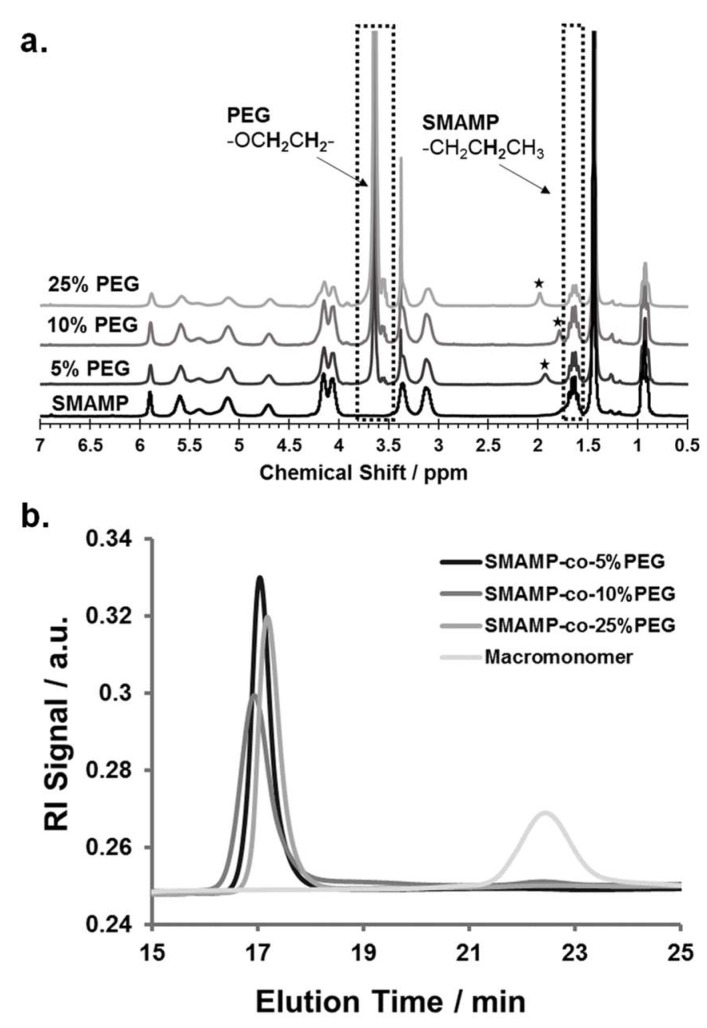
(**a**) ^1^H-NMR spectra of the three SMAMP-*co*-PEG copolymers (in CDCl_3_) with 5, 10, and 25 mol % PEG content. The PEG content was calculated by comparing the signal integral of the methylene protons from the SMAMP propyl chain at 1.63 ppm to the peak intensity of the ethylene glycol protons of the PEG repeat unit at 3.64 ppm. The asterisks (*) indicate water peaks. (**b**) GPC elugrams (in THF, calibrated with polystyrene standards) of SMAMP-*co*-PEG copolymers with 5, 10, and 25 mol % PEG content, compared to the GPC elugram of the macromonomer M.

The copolymers SMAMP-*co*-PEG were obtained by ring-opening metathesis polymerization (ROMP) using Grubbs' third generation catalyst (G3). The monomer P and the macromonomer M were copolymerized with different P:M ratios in dry tetrahydrofuran, so that copolymers with 5, 10, and 25 mol % PEG content were obtained (Scheme 1c; samples were named SMAMP-*co*-5%PEG, SMAMP-*co*-10%PEG, and SMAMP-*co*-25%PEG, respectively). The isolated yield after work-up by precipitation into diethyl ether/n-hexane was about 80%. The polymers thus obtained were analyzed by ^1^H-NMR spectroscopy (Figure 1a). Due to structural similarity, many peaks from the propyl SMAMP repeat unit and the PEG repeat unit overlapped. However, the characteristic peak at 3.64 ppm could be assigned to the methylene protons of the PEG component (OCH_2_CH_2_), while the peak at 1.63 ppm belonged to two propyl methylene protons of the SMAMP repeat unit (CH_2_CH_2_CH_3_). Thus, the PEG content of the copolymers could be determined by integrating and comparing these two signals. The data thus obtained is summarized in Table 2. The actual PEG content, as determined by NMR, was 4.5, 8.1, and 22.1% respectively. This closely matched the initial monomer feed ratio, demonstrating the high efficiency of the polymerization. The molar masses and molar mass distributions of the polymers were determined by gel-permeation chromatography (GPC, in THF using polystyrene standards). The GPC elugrams thus obtained are shown in Figure 1b, the analytical data are summarized in Table 2. The GPC peaks of the copolymers were symmetrical, monomodal, and elute significantly earlier than macromonomer M. This confirms that high molecular masses were obtained. While the target molecular mass for all polymers were 100,000 g mol^−1^, the calculated masses were significantly lower (66,000; 63,000; and 50,000 g mol^−1^, respectively). This can be explained by the different hydrodynamic volumes per unit mass of the comb polymers compared to the calibration standard: first, the chemical nature of the repeat units was different, and second and more importantly, the samples were highly branched. The polydispersity indices were 1.2 to 1.6 and thus a little higher than what would be expected for ROMP, however this can be attributed to one monomer being a macromonomer: these are generally more difficult to polymerize than conventional low molar mass monomers.

### 3.4. Synthesis and Physical Characterization of Surface-Attached Polymer Networks.

**Figure 2:**
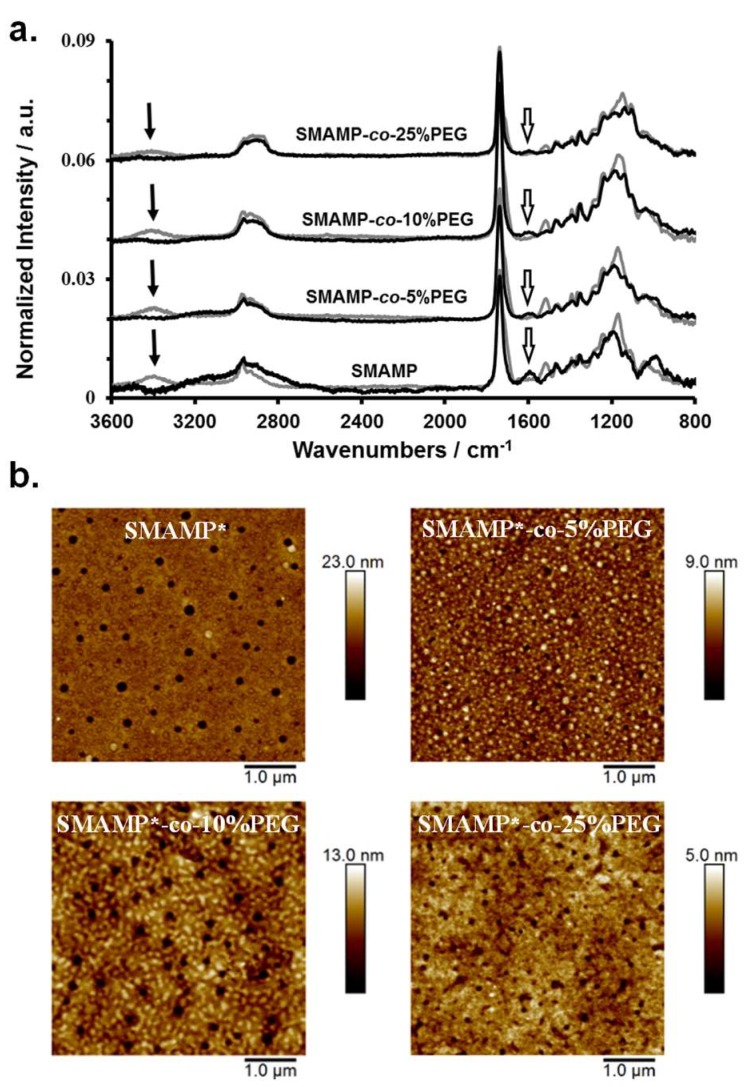
(**a**) FTIR spectra of the surface-attached SMAMP and SMAMP-*co*-PEG networks (grey lines), as well as the deprotected SMAMP* and SMAMP*-*co*-PEG networks (black lines). The black arrows indicate the signals of the Boc protective group of the SMAMP units, the open arrows designate the deprotected amine and ammonium groups. (**b**) AFM height images of the activated SMAMP*-*co*-PEG and SMAMP* networks (peak force tapping mode in air). The RMS roughness (Table 3) was calculated from these images using the Gwyddion software package.

To obtain bifunctional polymer surfaces with antimicrobial and protein-repellent moieties, the SMAMP-*co*-PEG polymers were surface-immobilized as networks (Scheme 1d), and then activated. For this, they were each dissolved in a mixture of CHCl_3_ and THF, to which the cross-linker pentaerythritoltetrakis(3-mercapto­propionate) (=tetrathiol) was added. This solution was spin-coated onto a solid substrate (either a silicon wafer or a gold substrate) that had been functionalized with benzophenone as reported previously [25,33]. Upon UV irradiation, the polymers were simultaneously cross-linked (by thiol-ene reaction of the polymer double bonds with the SH groups of the crosslinker) and surface-immobilized (by C,H insertion reactions between the surface-attached benzophenone groups and C-H bonds of the polymer). The samples were then washed with THF to remove unbound cross-linker and polymer chains. To remove the N-Boc protective group and thereby activate the antimicrobial function of the SMAMP repeat units, the surfaces were treated with hydrochloric acid. The resulting SMAMP*-*co*-PEG networks as well as their SMAMP-*co*-PEG precursors with the protective groups were characterized by ellipsometry, contact angle measurements, FTIR spectroscopy, atomic force microscopy (AFM), and photoelectron spectroscopy (XPS). The results of these measurements, compared to the data obtained for pure SMAMP networks (synthesized as described previously) [33], are summarized in Table 3. After deprotection, the thickness of the SMAMP*-*co*-PEG networks (determined by ellipsometry) decreased slightly (to 71 to 93 nm, Table 3) compared to the SMAMP-*co*-PEG networks, which due to the removal of the N-Boc protective groups. The hydrophilicity of the networks was investigated by static and dynamic water contact angles measurements. Overall and matching expectations, the contact angles of the protected and the deprotected networks decreased with increasing PEG content. Thus, the hydrophilicity of these surfaces increased with increasing content of hydrophilic PEG. The FTIR spectra of the protected and the deprotected networks are shown in Figure 2a. These data show that there is great structural similarity between the protected SMAMP-*co*-PEG networks (grey line) and their deprotected SMAMP*-*co*-PEG analogues (black lines). Both had the stretching vibration of C=O at 1732 cm^−1^. The stretching vibration of C–O was found at about 1140 and 1230 cm^−1^, and the C–H stretching vibrations of the aliphatic CH_3_ and CH_2_ groups were in the range of 2870 to 2950 cm^−1^. The spectra of the protected surfaces exhibited an additional characteristic peak at about 3400 cm^−1^, which was assigned to amide NH stretching vibration of the of N-Boc protective groups. This absorption band was weaker for the samples that had a higher PEG content and completely disappeared after acidic deprotection of the networks. Instead, a new absorption peak at about 1583 cm^−1^ was observed, which corresponds to the deformation vibration of the NH_2_ and NH_3_^+^ groups. Again, the intensity of this band decreased with higher PEG content, which is plausible as this corresponds to decreasing SMAMP content. Overall, these FTIR spectra confirmed the presence of the expected functional groups in the surface-attached SMAMP-*co*-PEG and SMAMP*-*co*-PEG copolymer networks, and indicated that the activation step (removal of the N-Boc group by HCl) was successful.

The morphology of the deprotected surface-attached SMAMP*-*co*-PEG networks was characterized by atomic force microscopy (AFM) using the peak force tapping mode. The resulting height images are shown in Figure 2b, together with a height image of SMAMP* for comparison. The SMAMP* network has the typical morphology obtained for surface-attached polymer networks that are cross-linked by an additional low molecular weight cross-linker [33,39]: small pores with a diameter of about 100 nm formed (previous areas of excess cross-linker), which are surrounded by a homogeneous polymer coating with a relatively low rms roughness (2.1 nm). These pores are also observed for the SMAMP*-*co*-PEG networks. The slightly different pore sizes in the various samples reflect the different polymer compositions, with a different overall hydrophilicity. This leads to a slightly different partition of the tetrathiol cross-linker between the polymer and the pure cross-linker microphases. Interestingly, the SMAMP*-*co*-PEG networks with 5 and 10 mol % (corresponding to about 19 to 32 mass % PEG) have further structural features. For the SMAMP*-*co*-5%PEG sample, small dots of PEG-rich domains in a SMAMP matrix can be seen. When the PEG content increased to 10 mol %, the PEG domains increased further, forming irregular wormlike patterns. For surfaces with 25 mol % PEG (corresponding to about 60 mass % PEG), the morphology appeared to be more homogeneous. Apparently, the PEG arms segregate to the surface and cover a large area of the polymer–air interface. It is well known that the highly mobile and hydrophilic PEG chains can easily rearrange on the surface depending on copolymer composition and environmental influences (e.g., humidity) [40,41]. 

The SMAMP* and SMAMP*-*co*-PEG networks were also analyzed by photoelectron spectroscopy (XPS). Using this method, the elemental composition on the top of few nanometers of each sample was probed. The XPS data obtained for carbon, nitrogen, and oxygen, as well as the elemental compositions of these of these polymers calculated from their molecular formulae are presented in Table 4. For all polymers, the carbon and oxygen contents determined by XPS were a little higher than the calculated data, yet there is no trend in that data that would indicate a preferred polymer orientation at the interface with increasing PEG content. The amount of nitrogen, however, decreases disproportionately in the XPS/calc. signal ratio with increasing PEG content. Thus, with increasing PEG content, the PEG chains seem to dominate the air-polymer interface, while SMAMP gets hidden underneath. This is in line with expectations since the PEG chains are longer and more flexible than the SMAMP repeat units. Thus, the XPS data complement the FTIR, ellipsometry, contact angle, and AFM results.

### 3.5. Protein Adhesion and Antimicrobial Activity of Surface-Attached Polymer Networks.

To verify that the bifunctional copolymer networks were indeed protein-repellent, their interaction with the protein fibrinogen was studied by surface plasmon resonance spectroscopy (SPR). For this, the SMAMP*-*co*-PEG networks and a pure SMAMP* reference network were surface-immobilized on gold substrates as described previously [25,31,33]. After each fabrication step, full angular reflectivity curves were recorded (see Figure S2). After that, the activated surfaces were exposed to fibrinogen (1 mg mL^−1^ in HEPES buffer). The interaction of this protein with the surfaces was monitored by SPR using the kinetics mode, where time-dependent changes in reflectivity at constant angle were measured at room temperature. Additionally, full angular reflectivity curves of the dry surfaces were recorded before and after the kinetics experiments to quantify the amount of irreversibly adhered protein. 

**Figure 3:**
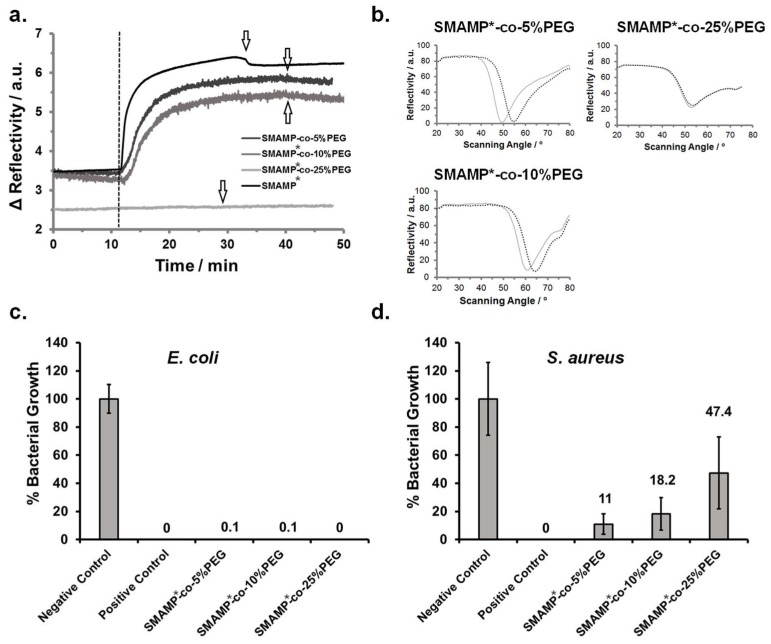
(**a**) Kinetics of fibrinogen adsorption on SMAMP*-*co*-PEG and SMAMP* monitored by surface plasmon resonance spectroscopy (SPR). (**b**) Full angular reflectivity SPR curves of the dry SMAMP*-*co*-PEG samples before and after protein adhesion. (**c**) and (**d**) Antimicrobial activity of the SMAMP*-*co*-PEG and SMAMP* surfaces against *E. coli* (**c**) and *S. aureus* (**d**). Bacterial growth (percentage of surviving colony forming units after 4 h incubation time, normalized to the negative control (=100% growth)) is shown for each material. For each data point, the assay was performed in duplicate, with five samples per repetition. The negative control was a blank silicon wafer piece, the positive control was a wafer piece to which chlorhexidine digluconate had been added.

In the kinetics experiment (Figure 3a), the surfaces were first exposed to buffer for about 10 min. The dashed line marks the time point of protein injection. If the reflectivity signal stays constant over time, no protein is adsorbed. If it increases, protein adheres to the surface and thereby changes the dielectric properties of the sample. For SMAMP*-*co*-25%PEG (light grey line), only very little protein adhesion was observed. For the other samples, protein adhesion was visible and increased with decreasing PEG content. This was confirmed by the full angular reflectivity scans recorded before and after protein adhesion (Figure 3b): For SMAMP*-*co*-25%PEG, the position of the plasmon peak was almost unchanged. For the other samples, its minimum shifted to higher angles. By simulating these curves using the Winspall software, the protein adhesion can be quantified. This data is summarized in Table 5. It shows that the PEG content on the SMAMP*-*co*-10%PEG surfaces was already too low to substantially suppress fibrinogen adhesion and to work against the adhesive forces exerted on the negatively charged protein molecules by the cationic SMAMP* groups. On the other hand, SMAMP*-*co*-25%PEG adsorbed only 0.99 ng fibrinogen per mm^2^. In this case, the PEG coverage on the surface was high enough to substantially screen the electrostatic attraction of SMAMP* ammonium cations. However, quantitative protein repellency was also not observed. 

The antimicrobial activity of the surface-attached SMAMP*-*co*-PEG networks was studied using the standardized airborne antimicrobial assay [35]. In this experiment, two pathogenic bacterial strains, Gram-negative *Escherichia coli* and Gram-positive *Staphylococcus aureus*, were sprayed onto the test surfaces. As reported previously [35], uncoated silicon wafers (negative control) and uncoated silicon wafers impregnated with chlorhexidine digluconate (positive control) were tested along with the polymer coated samples. Each surface was sprayed with bacterial suspensions containing 10^6^ bacteria per cm³ and incubated for four hours. After that, the surviving bacteria were transferred onto agar plates and cultivated, each surviving bacteria forming a colony. By counting these colonies, the antimicrobial activity could be quantified (Figure 3c,d). The assay was performed twice with five samples of each material type. The error bars are the standard deviation calculated from these data. The antimicrobial activity of each polymer surface was reported as bacterial growth (in percent) normalized to the negative control. Unexpected, all surface-attached SMAMP*-*co*-PEG networks, regardless of their PEG content, killed ≥99.9% of the adherent *E. coli* bacteria and were thus bactericidal. However, the polymer surfaces were significantly less effective against *S. aureus* bacteria—here, the killing efficiency decreased with increasing PEG content from 89% killing (11% growth) for the surfaces with 5 mol % PEG content, up to 52.6% killing (47.4% growth) for the surfaces with 25% PEG content. In contrast, the SMAMP* surface had excellent antimicrobial activity [33].

### 3.6. Discussion

By copolymerization of monomer P and macromonomer M, SMAMP-*co*-PEG polymers with different amounts of the protein-repellent PEG and the (masked, N-Boc proteced) antimicrobial SMAMP functionality were obtained. These polymers were immobilized to form surface-attached networks, and activated. Physical characterization data showed that the substrates used were homogeneously covered with the SMAMP*-*co*-PEG polymer, and that the PEG moieties tended to segregate to the polymer–air interface. Thus, with increasing PEG content, the interface became increasingly hydrophilic and protein-repellent, as demonstrated by the protein adhesion assay. However, 10 mol % (more than 30 mass %). PEG was still insufficient to effectively shield the materials from fibrinogen adhesion; for this, 25 mol % PEG were required. The antimicrobial activity of the SMAMP*-*co*-PEG polymers originates from the electrostatic interaction of the activated SMAMP* groups with the negatively charged cell envelope of bacteria, as reported previously [33]. However, the SMAMP*-*co*-PEG surfaces were only fully active against *E. coli*, while their activity against *S. aureus* was already compromised by as little as 5 mol % (18.8 mass %) PEG. The long PEG chains seem to prevent the close interaction of bacteria with the surface, and also might reduce the surface charge density. Kügler et al. found that the positive charge density of grafted poly(vinylpyridine) chains necessary for killing Gram-positive *Staphylococcus epidermidis* was 10 times higher than for Gram-negative *E. coli* [21]. In the data here presented, we also see that Gram-positive *S. aureus* is significantly less affected by the SMAMP* moieties than Gram-negative *E. coli*. Previous data from our group also showed that Gram-negative bacteria were more susceptible to SMAMP*-coated surfaces than Gram-positive ones [33]. Also, bifunctional polymer surfaces with SMAMP* patches and polyzwitterionic patches were more active against Gram-negative bacteria than against Gram-positive ones. In that data, we also saw that if the SMAMP patch size was too small, antimicrobial activity against *E. coli* became compromised [32]. Thus, the emerging picture for bifunctional polymer surfaces, whether from hierarchically organized copolymers or from microstructured surfaces, is that the overall local number of cationic groups interacting with each bacterial cell is crucial for the fate of that cell. This critical number seems to depend on the global charge density of the polymer itself (functional groups per nm^2^), the distance up to which these groups can be approached (i.e., whether direct surface access is blocked by PEG or polyzwitterion chains), and the electrostatic charge of the bacteria themselves. For example, it is known that *E. coli* bacteria approximately have a 5 times greater surface area per cell and an up to 15 times larger negative surface potential than *S. aureus* [42,43]. Thus, *E. coli* bacteria have more negative charges per cell available for electrostatic binding to the SMAMP moieties. In the case of the materials presented here, even though *E. coli* bacteria are larger than *S. aureus* bacteria, this negative potential enables *E. coli* to overcome the entropic barrier of the PEG groups and to sufficiently interact with the polymer surfaces to eventually get killed. On the other hand, it seems like the electrostatic interactions of the surfaces with *S. aureus* bacteria are not enough to overcome the shielding of the PEG moieties. This finding goes in line with observations reported by others: Fang et al. observed that a minimum cationic surface charge density of cationic functionality nanoparticles or polycations immobilized in a PEG brush was needed for *S. aureus* adhesion and antimicrobial activity; Cavallaro et al. showed that amine-coated surfaces had a specific threshold of surface-immobilized quaternary ammonium groups to induce significant antimicrobial effect against *E. coli*; and Gottenbos et al. reported that positively charged poly(methacrylate) surfaces showed a higher reduction of adhered viable counts for Gram-negative bacteria (*E. coli* and *P. aeruginosa*) than for Gram-positive bacteria (*S. aureus* and *S. epidermidis*), which is caused by weak electrostatic interaction with the thick bacterial cell membrane of Gram-positive bacteria [44,45,46].

It is always difficult to compare microbiological data from different laboratories because testing methods, controls used, and even environmental conditions may differ widely. Thus, it is not easy to say what is ‘the best’ bifunctional antimicrobial and protein-repellent coating currently known. However, a few trends become apparent. From the materials described in the introduction, the nisin-hyaluronic acid coating had up to 99.8% antibacterial activity against *S. epidermidis* after 3 h of contact with the bacterial suspension (at bacterial concentration about 10^8^ bacteria per cm³). However, this surface suffered from bacterial adhesion compared to the peptide-free surface [27]. The chitosan-poly(2-hydroxyethyl methacrylate) brushes had less than 10% protein adhesion, and killed up to 80% of *E. coli* bacteria after 4 h exposure to the bacterial suspension (bacterial concentration: about 10^6^ bacteria per cm³) [28].The polymer brushes with zwitterionic groups and quaternary ammonium functionalities had an increased protein adhesion, and killed 72% of *E. coli* bacteria after 3 h of exposure the surface to the bacterial suspension (at a bacterial concentration of 10^9^ bacteria per cm³) [29]. Thus, even though the here presented materials only have significant activity against *E. coli*, they seem to be at least comparable in their bioactivity profile to these reference polymers. Further work, however, needs to be dedicated to simultaneous protein-repellency and broad spectrum antimicrobial activity, not to mention simultaneous cell compatibility.

## 4. Conclusion

In this report, the synthesis and characterization of bifunctional surface-attached polymer networks containing protein-repellent PEG moieties and antimicrobial SMAMP* groups were presented. Varying the PEG content of these materials from 5–25 mol % had a profound effect on the interaction of these surfaces with bacteria and proteins. An optimal dual activity was obtained for the SMAMP*-*co*-25%PEG material, which had a protein repellency >92% (compared to a pure SMAMP surface), and quantitatively killed *E. coli*, but not *S. aureus* bacteria. Apparently, a higher local charge density is necessary to also successfully eliminate the Gram-positive bacteria, although other features such as loss of hydrophobicity [33] with increasing PEG content might also play a role. Thus, this study has provided general insight into understanding of how combining antimicrobial and protein-repellent functionalities can affect the bioactivity of the resulting bifunctional surface. Additionally, the presented SMAMP*-*co*-25%PEG networks could be useful coatings for urinary catheters, which often fail due to biofilms formation involving *E. coli*. For this, however, their long term stability and sterilizability have to be evaluated.

Only very recently, we were able to combine antimicrobial activity and protein-repellency in a single polymer component using a polyzwitterionic material [25,47]. While the exact mechanism of activity and the long term performance of this material are still being investigated, it seems that such a single component material might be an easier pathway to dual antimicrobial activity and protein repellency than finding the ‘sweet spot’ of the perfect balance of cationic and protein-repellent components in bifunctional polymer surfaces. However, each approach has its merits, and might be of different use in different fields of application. 

## Figures and Tables

**Table 1:** Reagent amounts for the synthesis of the SMAMP-*co*-PEG polymers.

Polymer	Monomer M		Monomer P		Catalyst G3		Solvent
	n/mmol	m/mg	n/mmol	m/mg	n/mmol	m/mg	mL
SMAMP*-*co*-5%PEG	0.07	116	1.36	500	5.7·10^−3^	4.2	7
SMAMP*-*co*-10%PEG	0.15	244	1.36	500	6.5·10^−3^	4.7	7
SMAMP*-*co*-25%PEG	0.45	733	1.36	500	9.5·10^−3^	6.9	8

**Table 2:** Characterization of the three SMAMP-*co*-PEG polymers. The PEG content determined from the ^1^H-NMR spectra was compared to the calculated values; the number average molar mass *M_n_* and the polydispersity index (*M_w_*/*M_n_*) were determined by gel permeation chromatography.

Copolymer	SMAMP to PEG Ratio	PEG Content				*M_n_*/kg mol^−1^	*M_w_*/*M_n_*
		mol %		mass %			
		calc.	NMR	calc.	NMR		
SMAMP-*co*-5%PEG	95:5	5	4.5	18.8	17.3	66	1.3
SMAMP-*co*-10%PEG	90:10	10	8.1	32.8	28.0	63	1.6
SMAMP-*co*-25%PEG	75:25	25	22.1	59.5	55.6	50	1.2

**Table 3:** Physical characterization data of SMAMP-*co*-PEG networks (protected) and SMAMP*-*co*-PEG networks (deprotected). The dry layer thickness was determined by ellipsometry; $\theta_{static}$, $\theta_{adv}$ and $\theta_{rec}\;$ = static, advancing and receding contact angles; rms roughness was determined by atomic force microscopy (AFM) from the images shown in Figure 2b.

	SMAMP	SMAMP*	SMAMP-*co*-5% PEG	SMAMP*-*co*-5% PEG	SMAMP-*co*-10% PEG	SMAMP*-*co*-10% PEG	SMAMP-*co*-25% PEG	SMAMP*-*co*-25% PEG
Thickness/nm	62 ± 2	53 ± 4	86 ± 2	79 ± 1	101 ± 2	93 ± 1	79 ± 2	71 ± 3
$\theta_{\mathrm{static}}$/°	82 ± 2	51 ± 1	89 ± 1	52 ± 2	85 ± 3	51 ± 2	79 ± 1	46 ± 2
$\theta_{\mathrm{adv}}$/°	91 ± 4	56 ± 2	90 ± 2	61 ± 2	87 ± 1	58 ± 3	85 ± 2	52 ± 1
$\theta_{\mathrm{rec}}$/°	43 ± 4	26 ± 2	42 ± 3	24 ± 2	41 ± 1	27 ± 1	37 ± 3	22 ± 2
Roughness/nm		2.1		1.1		1.9		0.6

**Table 4:** XPS data for the surface-attached SMAMP* and SMAMP*-*co*-PEG networks (deprotected), compared to the elemental composition of these polymers calculated from their respective molecular structures.

Polymer	Elemental Composition/%						Ratio XPS/calc.		
	XPS			calc.					
	C 1s	N 1s	O 1s	C	N	O	C	N	O
SMAMP*	71.2	3.2	25.6	68.4	5.3	26.3	1.04	0.61	1.02
SMAMP*-*co*-5%PEG	70.6	3.1	26.3	68.3	5.0	26.7	1.03	0.62	1.02
SMAMP*-*co*-10%PEG	70.1	2.8	27.1	68.2	4.7	27.0	1.03	0.38	1.03
SMAMP*-*co*-25% PEG	70.5	1.3	28.2	68.0	3.9	28.1	1.04	0.33	1.04

**Table 5:** Bioactivity data of SMAMP*-*co*-PEG samples and the SMAMP* reference surface: fibrinogen adhesion (in ng mm^−2^), determined by surface plasmon resonance spectroscopy, and antimicrobial activity (% bacterial growth) against *E. coli* and *S. aureus* bacteria.

Polymer	Protein Adhesion/ng mm^−2^	Antimicrobial Activity/% growth	
		*E. coli*	*S. aureus*
SMAMP*	11.3	-	-
SMAMP*-*co*-5%PEG	9.8	0.1	11
SMAMP*-*co*-10%PEG	8.4	0.1	18.2
SMAMP*-*co*-25% PEG	0.99	0	47.4

## Supplementary Materials

The following are available online at http://www.mdpi.com/1996-1944/11/8/1411/s1, Figure S1: Characterization of the PEG macromonomer, Figure S2: Protein adhesion study.
