# Supplementary material for: Surface-Attached Poly(oxanorbornene) Hydrogels with Antimicrobial and Protein-Repellent Moieties: The Quest for Simultaneous Dual Activity
Source: Materials (Basel). 2018 Aug 11;11(8):1411. doi: 10.3390/ma11081411 (PMC6120009; doi:10.3390/ma11081411)
Supplement: Supplementary file 1 [file materials-11-01411-s001.pdf]

Article

Surface-attached Poly(oxanorbornene) Hydrogels With Antimicrobial and Protein-repellent Moieties: The Quest For Simultaneous Dual Activity

Monika Kurowska, Vania T. Widyaya, Ali Al-Ahmad, and Karen Lienkamp*

Freiburg Center für Interactive Materials and Bioinspired Technologies (FIT) and Department of Microsystems Engineering (IMTEK), Albert-Ludwigs-Universität, Georges-Köhler-Allee 105, 79110 Freiburg, Germany; monika.kurowsa@imtek.uni-freiburg.de (M.K.); vania.widyaya@imtek.uni-freiburg.de (V.T.W.), ali.al-ahmad@uniklinik-freiburg.de (A.A.);

*Correspondence: lienkamp@imtek.uni-freiburg.de (K.L.); Tel.: +49 761 203 95090

Characterization of PEG macromonomer

a.

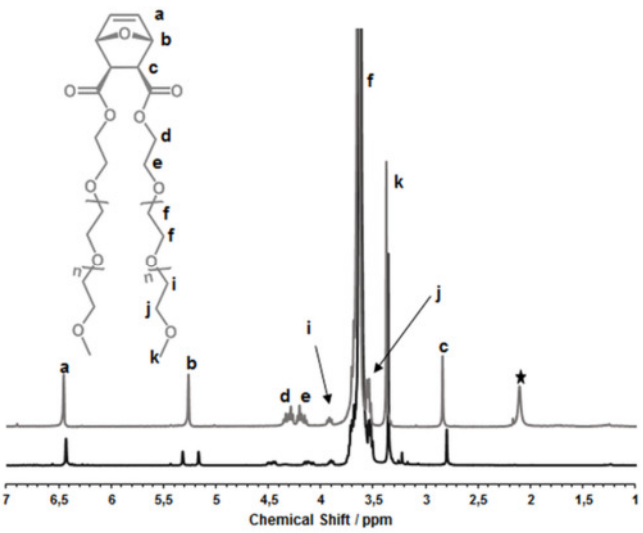

b.

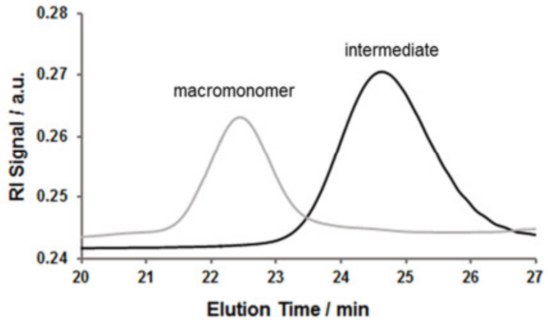

c.

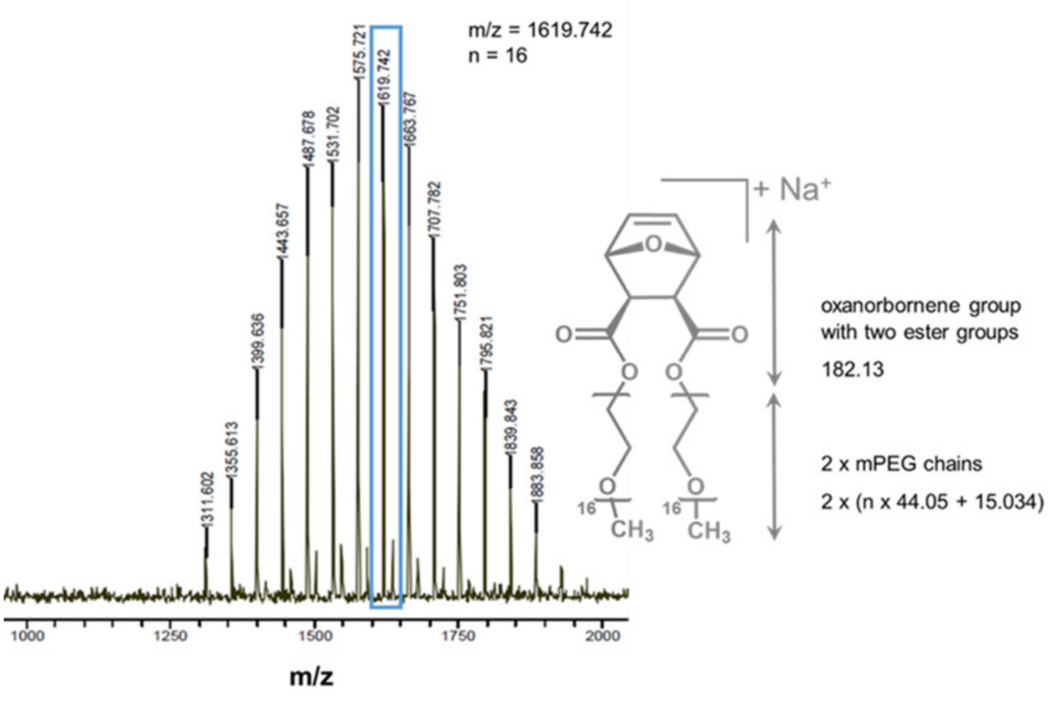

Figure S1: **a.** ^{1}HNMR spectrum of the intermediate **3** (bottom) and the PEG macromonomer **M** in $CDCl_3$. The peaks at 5.32 and 5.17 ppm, which correspond to protons of the intermediate with one PEG chain, fuse in one peak b after the second mPEG arm is attached. This confirms the quantitative functionalization of the macromonomer. The asterisks (*) indicate water peaks. **b.** GPC traces of the PEG macromonomer **M** (left) and its intermediate **3** (right). As expected, macromonomer eluted faster due to the higher molecular weight. GPC was measured in THF, calibrated with PS standards. **c.** MALDI-TOF spectrum of macromonomer **M** (matrix: DCTB in chloroform $CHCl_3$, counterion: Na^+). The peak labeled at 1619 Da corresponds to the molecular weight of the macromonomer with two PEG chains with on average 16 repeat units.

Protein adhesion study

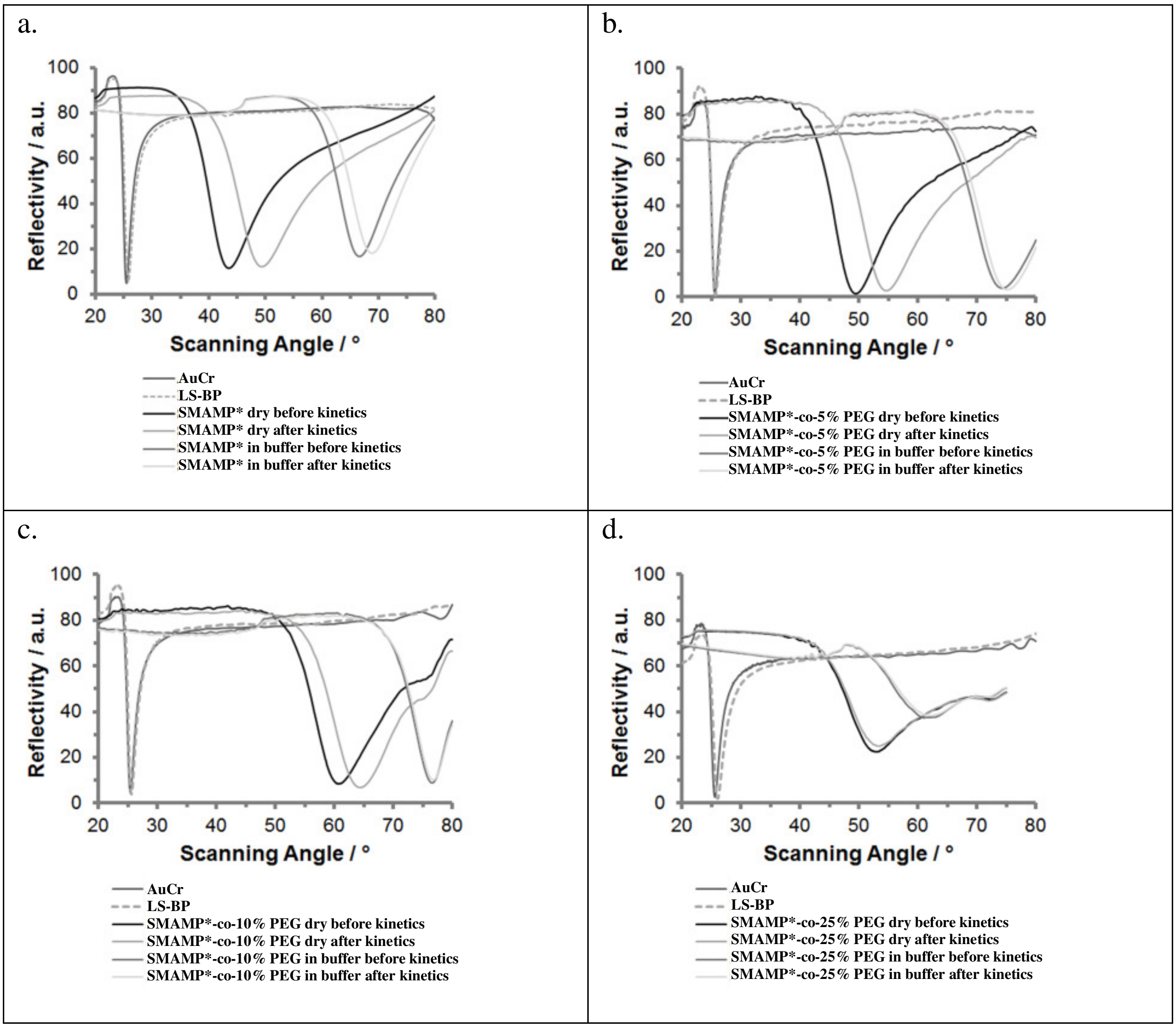

Figure S2: Fibrinogen adsorption on the surface-attached **SMAMP*** (a.), **SMAMP*-*co*-5%PEG** (b.), **SMAMP*-*co*-10%PEG** (c.) and **SMAMP*-*co*-25%PEG** networks studied by SPR. SPR angular reflectivity curves were measured for a gold substrate and after each modification step (LS-BP functionalization, deprotection of surface-attached SMAMP and SMAMP-*co*-PEG networks). Additionally, angular reflectivity scans before and after kinetic of fibrinogen adhesion in HEPES buffer (10 mM, 154 mM NaCl, pH 7.4) and in dry state (against nitrogen flow) are shown.
